# Molecular markers of prognosis in canine cortisol‐secreting adrenocortical tumours

**DOI:** 10.1111/vco.12521

**Published:** 2019-08-04

**Authors:** Karin Sanders, Gerjanne J. van Staalduinen, Maarten C. M. Uijens, Jan A. Mol, Erik Teske, Adri Slob, Jan Willem Hesselink, Hans S. Kooistra, Sara Galac

**Affiliations:** ^1^ Department of Clinical Sciences of Companion Animals, Faculty of Veterinary Medicine Utrecht University Utrecht the Netherlands

**Keywords:** adrenocortical adenoma, adrenocortical carcinoma, cancer, canine, Cushing syndrome, prognostic, treatment targets

## Abstract

Hypercortisolism is caused by a cortisol‐secreting adrenocortical tumour (ACT) in approximately 15%‐20% of cases in dogs. Little is known about which molecular markers are associated with malignant behaviour of canine ACTs. The objective of this study was to identify molecular markers of prognosis, which could be useful to refine prognostic prediction and to identify potential treatment targets. Cortisol‐secreting ACTs were included from 40 dogs, of which follow‐up information was available. The ACTs were classified as low risk of recurrence tumours (LRT; n = 14) or moderate‐high risk of recurrence tumours (MHRT; n = 26), based on the novel histopathological Utrecht score. Normal adrenals (NAs) were included from 11 healthy dogs as reference material. The mRNA expression of 14 candidate genes was analysed in the 40 ACTs and in 11 NAs with quantitative RT‐PCR. The genes' expression levels were statistically compared between NAs, LRTs and MHRTs. Univariate and multivariate analyses were performed to determine the association of the genes' expression levels with survival. Seven genes were differentially expressed between NAs and ACTs, of which pituitary tumour‐transforming gene‐1 (*PTTG1*) and topoisomerase II alpha (*TOP2A*) were also differentially expressed between LRTs and MHRTs. In survival analyses, high expression levels of Steroidogenic factor‐1 (*SF‐1*), *PTTG1* and *TOP2A* were significantly associated with poor survival. In conclusion, we have identified several genes that are part of the molecular signature of malignancy in canine ACTs. These findings can be used to refine prognostic prediction, but also offer insights for future studies on druggable targets.

## 1 INTRODUCTION

Spontaneous hypercortisolism is one of the most common endocrine disorders in dogs, with an estimated prevalence of approximately 1 in 400.^1^ It is caused by a cortisol‐secreting adrenocortical tumour (ACT) in 15% to 20% of cases. If no metastases are detectable, the treatment of choice for an ACT is adrenalectomy. Other or adjunctive treatment options include the steroidogenesis inhibitor trilostane, which will only reduce the clinical signs of hypercortisolism and has no effect on tumour growth, and the adrenocorticolytic agent mitotane, which can cause considerable side‐effects.^2^

Reported recurrence rates after adrenalectomy vary between 12% and 38%,^3^, ^4^, ^5^ which can be caused by metastases or regrowth of the ACT. Assessing the risk of recurrence after adrenalectomy is usually based on histopathology. However, the histopathological parameters that are mostly used in the assessment of canine ACTs^6^ often have high interobserver variability in human ACTs,^7^, ^8^ and some studies did not observe a significant difference in survival times of dogs after adrenalectomy based on their histopathological diagnosis.^4^, ^9^ To improve the reliability and prognostic value of histopathology in canine cortisol‐secreting ACTs, we recently introduced a new histopathological scoring system: the Utrecht score.^5^ The Utrecht score was based on parameters with low intra‐ and interobserver variability and their association with the dogs' survival times. It includes assessment of the Ki67 proliferation index, the presence of necrosis, and the percentage of clear/vacuolated cytoplasm, and increasing Utrecht scores were significantly associated with shorter survival times.^5^

In the most recent study on ACTs, the median survival time of 19 dogs with recurrence was 16.9 months (95% CI 10.8‐49.3 months).^5^ If these dogs would have been classified as having a high risk of recurrence, they could have received adjuvant treatment post‐operatively which might have improved their survival times. Moreover, if molecular markers that are associated with malignancy and thus a high risk of recurrence could be identified, this could give more insight into which molecular pathways are useful to target for future treatment options. However, at present, little is known about which molecular markers are associated with malignancy of canine ACTs. Previous research by our group showed that the mRNA expression of Steroidogenic factor‐1 (SF‐1; NR5A1), an important regulator of adrenal development and steroidogenesis, was significantly higher in ACTs of dogs that had recurrence of hypercortisolism within 2.5 years after adrenalectomy, than in ACTs of dogs that had no recurrence for at least 2.5 years.^10^ High expression of SF‐1 is also an important negative prognostic indicator in human ACTs.^11^, ^12^ Other molecular markers that have prognostic value in human ACTs include pituitary tumour‐transforming gene‐1 (PTTG1),^13^ topoisomerase II alpha (TOP2A),^14^ Vav Guanine Nucleotide Exchange Factor 2 (VAV2)^15^ and (for childhood ACTs) B‐cell lymphoma 2 (BCL2).^16^

To identify molecular markers of malignancy in canine ACTs, we used a candidate gene approach. The genes we selected are involved in adrenal‐specific pathways, proliferation/apoptosis‐related pathways, or both, and were selected based on an estimation of their prognostic relevance and whether they or the pathways they are involved in are potential druggable targets. The results of this study could be used to refine prognostic classification and could provide more insight into which genes or pathways are interesting for future studies on new treatment options. To this end, we evaluated the mRNA expression of 14 candidate genes and assessed whether they were associated with the histopathological diagnosis of the ACT based on the Utrecht score, and/or with the dogs' survival times.

## 2 MATERIALS AND METHODS

### 2.1 Case selection

Cortisol‐secreting ACTs of dogs were collected between 2002 and 2015. All dog owners gave permission to use the ACT tissue for research purposes. Non‐suppressible hypercortisolism and the presence of an enlarged adrenal gland was detected as described previously.^5^ All dogs underwent unilateral adrenalectomy, which was performed by one of in total four experienced veterinary surgeons. Dogs were excluded from the study when they had bilateral adrenal tumours; when no formalin‐fixed paraffin‐embedded tissue and snap‐frozen material was available; when they were euthanized or died before, during, or within 2 weeks after adrenalectomy; and when less than 3 months of follow‐up information was available. All dogs with ACTs in this study were also included in the previously published series where we introduced the Utrecht score.^5^ The adrenal glands of 11 healthy dogs were used as reference material; these dogs were euthanized for reasons unrelated to this study which was approved by the Ethical Committee of Utrecht University conform Dutch legislation.

### 2.2 Histopathological evaluation

The histopathological evaluation of each ACT was performed as described previously.^5^ Formalin‐fixed paraffin‐embedded tissues were cut in 4 μm thick sections on Superfrost Plus Adhesion Microscope Slides (Thermo Fisher Scientific, Breda, The Netherlands). The tissue sections were stained with haematoxylin and eosin, and immunohistochemical staining for Ki67 (MIB‐1 clone, M7240, Dako, Agilent, Amstelveen, The Netherlands) was performed as described previously.^5^ All ACTs were evaluated by two observers. For each ACT, the Utrecht score was calculated: the Ki67 proliferation index +4 if ≥33% of cells have clear/vacuolated cytoplasm, and + 3 if necrosis was present. The ACTs were classified according to their Utrecht score as low risk of recurrence tumours (LRTs; < 6 in Utrecht score) or moderate‐high risk of recurrence tumours (MHRTs; ≥ 6 in Utrecht score).

### 2.3 Quantitative RT‐PCR

Tissues were snap‐frozen within 10 minutes after adrenalectomy (ACTs) or euthanasia (normal adrenals; NAs). RNA was isolated from tumour tissue or from the adrenal cortex of NAs with the RNeasy Mini Kit (Qiagen, Venlo, The Netherlands) according to the manufacturer's instructions. The RNA concentrations were measured with Nanodrop (ND‐1000; Isogen Life Sciences, Utrecht, The Netherlands) and cDNA was subsequently synthesized with the iScript cDNA Synthesis Kit (Bio‐Rad, Veenendaal, The Netherlands) according to the manufacturer's instructions, and diluted to 1 ng/μL.

Using SYBR‐green Supermix (Bio‐Rad) and a CFX384 Touch Real‐Time PCR Detection System (Bio‐Rad), the mRNA expression levels of 14 genes were analysed with quantitative RT‐PCR (RT‐qPCR) analysis: melanocortin 2 receptor (*MC2R*), inhibin alpha subunit (*INHA*), *SF‐1*, *VAV2*, pre‐B‐cell leukaemia transcription factor 1 (*PBX1*), pantetheinase (*VNN1*), and sterol O‐acyltransferase (*SOAT1*) (adrenal‐associated genes); and *PTTG1*, ribonucleoside‐diphosphate reductase subunit M2 (*RRM2*), *TOP2A*, *MKI67*, cyclin D1 (*CCND1*), Ras‐related C3 botulinum toxin substrate 1 (*RAC1*) and *BCL2* (proliferation‐ or apoptosis‐related genes). Primers (Table 1) were designed using Perl‐primer software,^17^ checked for secondary structure formation with the Mfold web server,^18^ and ordered from Eurogentec (Maastricht, The Netherlands). Optimization and primer specificity confirmation were performed as described previously.^19^

**Table 1:** Primer pairs

Target gene	Primer sequence (5′ ➔ 3′)	Accession number	Position	Annealing Tm (°C)
*MC2R*	Fw: TCA TGT GGT TTT GCC GGA AGA GAT	XM_022416941.1	296‐434	58.5
	Rv: AAT GGC CAG GCT GCA AAT GAA A			
*INHA*	Fw: AGG AGG ATG TCT CCC AGG C	XM_545660.6	331‐505	67.0
	Rv: GTG TGG AAC CAC AGG TGG GC			
*SF‐1*	Fw: AGG GCT GCA AGG GGT TTT TCA A	XM_846937.2	200‐342	59.0
	Rv: CAT CCC CAC TGT CAG GCA CTT CT			
*VAV2*	Fw: CTG CTT ACT GGA GAT TCA GG	XM_022423933.1	578‐654	58.0
	Rv: GGG TCA TGT AGT TCT TCT CG			
*PBX1*	Fw: GCA TCA GTG CTA ATG GAG GT	XM_022415304.1	1192‐1286	60.3
	Rv: GCA GGT ATC AGA GTG AAC ACT G			
*VNN1*	Fw: AGT TGA AAC TGC TTC TAC C	NM_001003372.1	902‐1028	61.6
	Rv: ACT TGA CAC CTG AAA TTC TC			
*SOAT1*	Fw: CAA CTA TCC TAG GAC TCC CAG	XM_005622465.3	986‐1150	60.3
	Rv: CAT AGG ACC AGA ACG CGA			
*PTTG1*	Fw: GCC TCA GAT GAC ACC TAT CCA G	XM_536445.5	457‐606	63.5
	Rv: AAG TTC CCT CTC CTC ATC AAG G			
*RRM2*	Fw: GAA GCT ACC TAT GGA GAA CGG	XM_540076.6	704‐890	61.5
	Rv: GGT GTT TGA ACA TCA GGC AG			
*TOP2A*	Fw: CGG ACA CCT ACA TTG GCT	XM_537646.5	277‐415	64.5
	Rv: GCA GCA TTG ACC AGA ATC TC			
*MKI67*	Fw: TCA GTT CCA GCA ATC CGA	XM_022411692.1	467‐643	61.5
	Rv: GCA GAG ATT CCT GTT TGC G			
*CCND1*	Fw: ACT ACC TGG ACC GCT	NM_001005757.1	410‐560	61.0
	Rv: CGG ATG GAG TTG TCA			
*RAC1*	Fw: TCC CTT ATC CTA TCC GCA AA	NM_001003274.2	204‐332	58.0
	Rv: ATG ATA GGG GTG TTG GGA CA			
*BCL2*	Fw: GGA TGA CTG AGT ACC TGA ACC	NM_001002949.1	959‐1039	62.0
	Rv: CGT ACA GTT CCA CAA AGG C			

*Note*: Primer pairs for RT‐qPCR analysis.

Abbreviations: Fw, forward primer; Rv, reverse primer.

For data normalization, the mRNA expression levels of five reference genes were analysed: signal recognition particle receptor, succinate dehydrogenase complex subunit A, ribosomal protein S5, hypoxanthine‐guanine phosphoribosyltransferase and tyrosine 3‐monooxygenase/tryptophan 5‐monooxygenase activation protein zeta.^20^, ^21^, ^22^

Two technical replicates were analysed for each sample. To exclude interference of genomic DNA, for each sample a control sample without reverse transcriptase was analysed. GeNorm software^23^ was used to analyse the reference genes' expression levels, which justified their use. The 2^−ΔΔCt^ method^24^ was used to calculate the normalized relative expression of each target gene.

### 2.4 Statistical analyses

Because the data were not normally distributed, which was observed using the Shapiro‐Wilk test, differences in mRNA expression levels between groups (NAs, LRTs, MHRTs) were analysed with the Kruskal‐Wallis test. Significant differences were analysed post hoc with the Mann‐Whitney *U*‐test with Bonferroni correction. Correlations between the Utrecht score and the gene mRNA expression levels were assessed using the Spearman's Rank Order Correlation.

For survival analyses, the ACT was considered to be the cause of death when the dog was euthanized due to recurrence of hypercortisolism, resulting from metastases or regrowth of the ACT. Survival times were recorded as the time between adrenalectomy and euthanasia due to recurrence (event occurred), or the time between adrenalectomy and the time of censoring (event did not occur). Dogs were censored when they died from an unrelated cause, were still alive at the end of the study, or were lost to follow‐up. The Cox proportional hazards model was used for univariate survival analyses. All variables with a *P*‐value of <.15 in univariate analyses were included in multivariate stepwise regression with forward selection.

To calculate optimal cut‐off values, receiver operating characteristic (ROC) curves were used. To obtain clear results, only dogs with evidently good or bad prognoses were included for ROC curves: dogs that had recurrence and were euthanized within 30 months after adrenalectomy were included in the positive group (n = 9), and dogs that had no recorded recurrence and lived for at least 30 months after adrenalectomy were included in the negative group (n = 13). The cut‐off value with the highest Youden index (sensitivity + specificity ‐ 1) was selected as the optimal cut‐off value. Survival times were subsequently calculated using the Kaplan‐Meier product‐limit method, and the log‐rank test was used to determine whether a difference in survival times between groups was significant.

*P*‐values <.05 were considered significant. All statistical analyses were performed with SPSS Statistics for Windows (Version 24.0, IBM Corp, Armonk, New York).

## 3 RESULTS

### 3.1 Cases

Clinical data of the dogs included in this study are described in Table S1. Based on histopathological analyses using the Utrecht score, 14 (35%) ACTs were classified as LRTs (Utrecht scores range: 0.4‐5.6) and 26 (65%) as MHRTs (Utrecht scores range: 6.1‐29.2). The median survival time was not reached for the LRT group (recurrence observed in 1/14 dogs), and was 49.3 months (95% CI 12.9‐85.6 months) for the MHRT group (recurrence observed in 14/26 dogs).

### 3.2 Differential expression

**Figure 1:**
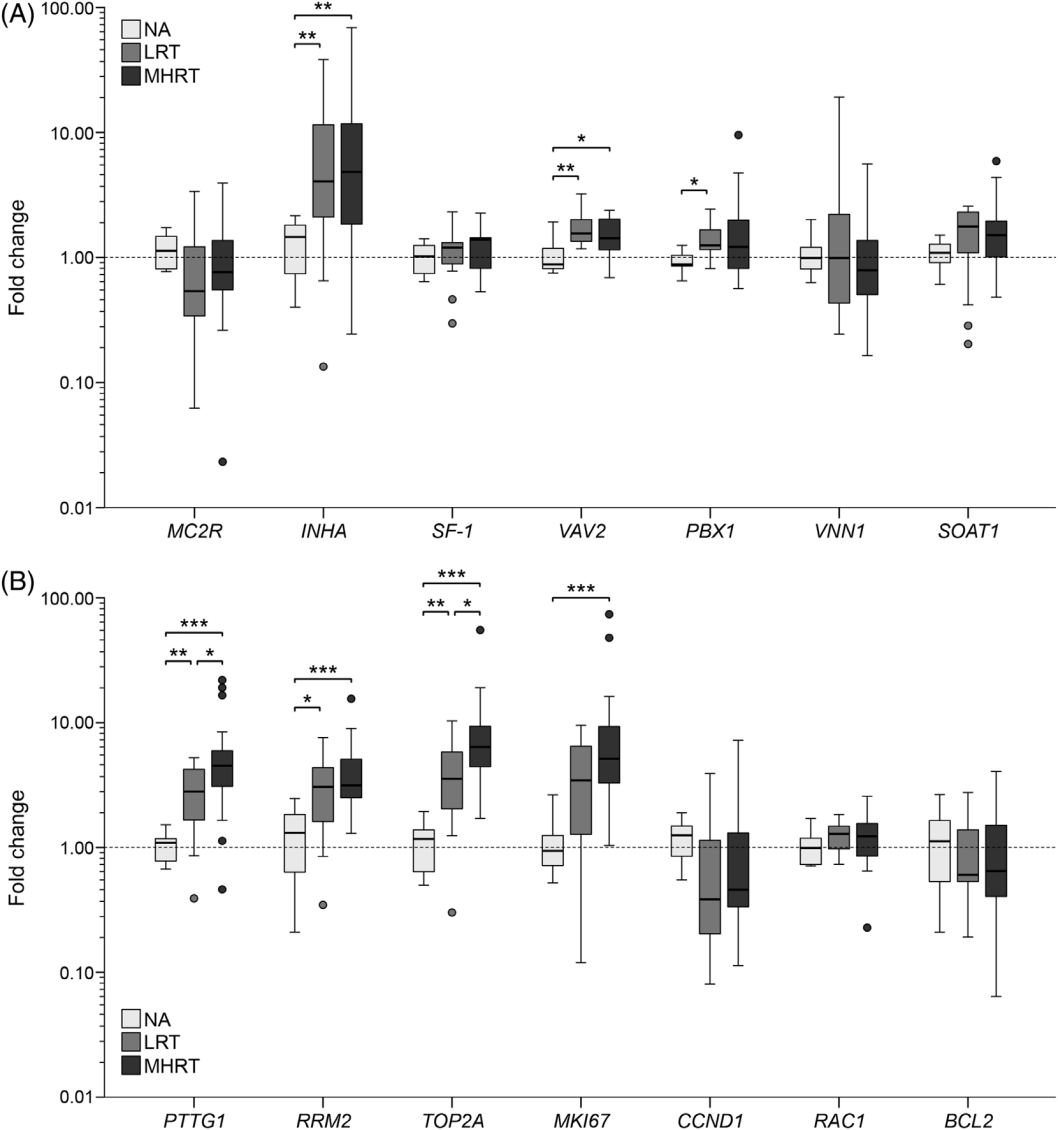
The mRNA expression levels in fold change (relative to the mean NA expression) of seven adrenal‐associated genes (A) and seven proliferation‐ or apoptosis‐associated genes (B). The dotted line represents the mean fold change of the NAs. The circles above and below the box plots indicate outliers. *P < .05, **P < .01, ***P < .001. NA, normal adrenal (n = 11); LRT, low risk of recurrence tumour (n = 14); MHRT, moderate‐high risk of recurrence tumour (n = 26)

In comparing the mRNA expression levels of the 14 candidate genes between NAs, LRTs, and MHRTs, 7 genes showed significant differences: *INHA*, *VAV2*, *PBX1*, *PTTG1*, *RRM2*, *TOP2A* and *MKI67*. Of these genes, the mRNA expression levels (Figure 1) were significantly higher in LRTs compared to NAs for *PBX1*; in MHRTs compared to NAs for *MKI67*; in LRTs and in MHRTs compared to NAs for *INHA*, *VAV2* and *RRM2*; and in LRTs and MHRTs compared to NAs but also in MHRTs compared to LRTs for *PTTG1* and *TOP2A*. The mRNA expression levels of *PTTG1* and *TOP2A*, but not of the other 12 genes, were also significantly correlated with the Utrecht score as a continuous variable (Table S2).

### 3.3 Survival analyses

**Figure 2:**
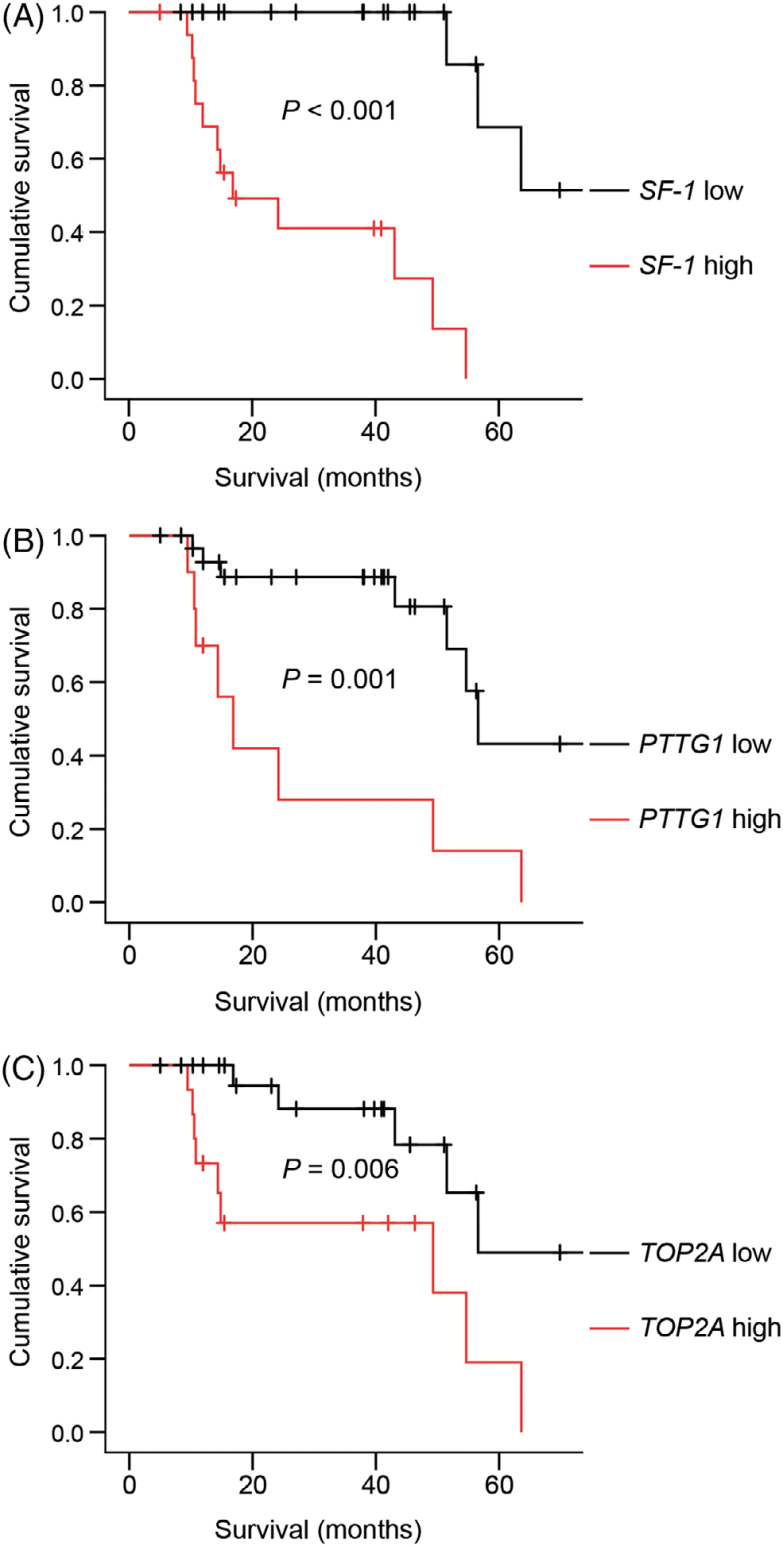
Survival stratified according to SF‐1 (A), PTTG1 (B) and TOP2A (C) mRNA expression using Kaplan–Meier analyses. Dogs were classified as having (A) low (fold change <1.35, n = 23) or high (≥ 1.35, n = 17) *SF‐1* expression; (B) low (<5.35, n = 30) or high (≥5.35, n = 10) *PTTG1* expression and (C) low (< 6.31, n = 24) or high (≥6.31, n = 15) *TOP2A* expression. Tick marks indicate censored dogs. *P*‐values indicate the significance of the difference between the respective groups as calculated with the log‐rank test. PTTG1, pituitary tumour‐transforming gene‐1; TOP2A, topoisomerase II alpha [Colour figure can be viewed at http://wileyonlinelibrary.com]

In assessing the genes' association with survival times, the mRNA expression levels of three genes were significant: *SF‐1*, *PTTG1* and *TOP2A* (Table 2). When we classified the ACTs based on the genes' optimal cut‐off values as calculated using ROC curves, the survival times were significantly different between the groups with high and low expression for all three genes (Figure 2). For *SF‐1*, the estimated median survival time of the group with low expression (fold change <1.35; n = 23) was not reached, and of the group with high expression (≥1.35; n = 17) 16.9 months (95% CI, 1.5‐32.2 months) (*P* < .001). For *PTTG1*, the estimated median survival time of the group with low expression (<5.35; n = 30) was 56.6 months (95% CI, 52.1‐61.1 months), and of the group with high expression (≥5.35; n = 10) 16.9 months (10.7‐23.0 months) (*P* = .001). For *TOP2A*, the estimated median survival time of the group with low expression (<6.31; n = 24) was not reached, and of the group with high expression (≥6.31; n = 15) 49.3 months (95% CI, 47.6‐61.7 months) (*P* = .006).

**Table 2:** Survival analyses

Gene	Hazard ratio (95%CI)	*P*‐value
*MC2R*	1.29 (0.77‐2.16)	.328
*INHA*	1.00 (0.96‐1.03)	.771
*SF‐1*	8.23 (2.43‐27.89)	.001*
*VAV2*	0.74 (0.27‐2.08)	.572
*PBX1*	1.14 (0.94‐1.38)	.187
*VNN1*	0.73 (0.44‐1.23)	.236
*SOAT1*	1.21 (0.85‐1.72)	.287
*PTTG1*	1.23 (1.11‐1.37)	<.001*
*RRM2*	1.10 (0.92‐1.32)	.276
*TOP2A*	1.06 (1.02‐1.11)	.005*
*MKI67*	1.02 (1.00‐1.05)	.080
*CCND1*	1.19 (0.82‐1.72)	.369
*RAC1*	2.65 (0.62‐11.24)	.188
*BCL2*	0.88 (0.47‐1.65)	.698

*Note*: Univariate analyses performed with the Cox proportional hazards model. Significant *P*‐values are indicated in italic font with an asterisk.

In multivariate analysis, the *SF‐1* mRNA expression (*P* = .001) and the Utrecht score (*P* < .001) were identified as independent predictors of survival.

## 4 DISCUSSION

In this study we identified seven genes that were differentially expressed between NAs and ACTs, of which *PTTG1* and *TOP2A* were also differentially expressed between LRTs and MHRTs. Moreover, we identified three genes of which high mRNA expression was significantly associated with poor survival: *SF‐1*, *PTTG1* and *TOP2A*. The prognostic relevance of these genes indicates that they could be useful to refine prognostic classification, but also identifies them as potential treatment targets.

SF‐1 is an orphan nuclear receptor that is important in both adrenal development and steroidogenesis,^25^ and the importance of *SF‐1* gene dosage in adrenocortical tumorigenesis has been shown in multiple studies.^11^, ^26^, ^27^ Interestingly, *SF‐1* expression did not differ between LRTs and MHRTs in the current study, nor between adrenocortical adenomas (ACAs) and adrenocortical carcinomas (ACCs) in a previous study,^10^ which might appear to contradict its prognostic relevance. However, this is remarkably similar to the SF‐1 expression pattern in human ACTs.^11^ This apparent contradiction could be related to the difference in SF‐1 function depending on the cellular context: in differentiated adrenocortical cells SF‐1 mostly stimulates hormone production, whereas in foetal adrenal development SF‐1 stimulates adrenal growth.^25^, ^28^ Possibly, ACA/LRT cells more closely resemble differentiated cells, and ACC/MHRT cells more closely resemble foetal cells, in which high SF‐1 expression might provide a specific growth advantage.^11^ Regardless of the mechanism, these results indicate that there could be room for improvement in the Utrecht score to refine prognostic classification. This was confirmed by the multivariate survival analysis, which showed that the Utrecht score and the *SF‐1* mRNA expression are independent predictors of survival. Because the range of *SF‐1* mRNA expression is relatively small, assessment of the *SF‐1* mRNA expression would not be feasible to include in routine diagnostic procedures. Other techniques such as immunohistochemistry to evaluate the SF‐1 protein expression could be analysed to determine whether this can improve prognostication.

The prognostic relevance of SF‐1 makes it an interesting therapeutic target. Compounds that can target SF‐1 activity, called SF‐1 inverse agonists, have been identified^29^, ^30^ and inhibited cell proliferation and steroid hormone production in vitro in human ACC cells.^31^ We showed in a previous study that one SF‐1 inverse agonist, compound #31, effectively inhibited cortisol production and SF‐1 target gene expression in canine adrenocortical cells in vitro.^32^ If SF‐1 inverse agonists will be further developed for clinical use, this may have much potential to improve the prognosis of dogs with an ACC with high SF‐1 expression.

PTTG1 is a securin that regulates sister chromatin separation during mitosis, and it plays a role in DNA repair, metabolism, senescence, apoptosis and gene transcription.^33^, ^34^ *PTTG1* is also a prognostic marker in human ACCs,^13^, ^35^ as well as in other tumour types in humans and dogs.^36^, ^37^, ^38^ Several drugs have been shown to inhibit PTTG1 expression, including BRAF, HDAC, Hsp90 and STAT3 inhibitors,^39^, ^40^, ^41^ which could be interesting options to target PTTG1 in canine ACTs.

TOP2A is a nuclear enzyme that facilitates DNA unlinking, which is required for DNA replication and chromosome segragation.^42^, ^43^ TOP2A is predominantly associated with proliferating cells, which makes it an interesting therapeutic target in cancer. *TOP2A* is also a prognostic marker in human ACCs and other tumour types.^14^, ^37^ Several TOP2A inhibitors are therefore successfully used in the clinic as anticancer drugs.^42^, ^43^ The chemotherapy protocol that is most effective in human ACCs is the combination of etoposide, doxorubicin and cisplatin with mitotane (EDP‐M).^44^ Of these, etoposide and doxorubicin are topoisomerase II inhibitors,^45^ and a recent study showed that the level of *TOP2A* expression in human ACCs is predictive of the response to the EDP‐M chemotherapy protocol.^14^ In view of the prognostic relevance of *TOP2A* in canine ACTs, topoisomerase II inhibitors could be an interesting treatment to improve the prognosis of dogs with high intra‐tumoural *TOP2A* expression.

The prognostic relevance of genes such as *VAV2, PBX1* and *RRM2* could have been underestimated in this study due to the relatively low number of cases, and it might be interesting to reanalyse the expression of these genes in a larger dataset. Moreover, this study was based on a candidate gene approach. Making use of for example RNA sequencing would be an interesting approach to identify prognostically relevant genes in an unbiased fashion.

In conclusion, in this study we have identified several genes that are part of the molecular signature of malignancy in canine ACTs. Most apparent molecular markers of prognosis were *SF‐1*, *PTTG1* and *TOP2A*. These findings can be used to refine prognostic prediction, but also offer substrate for future studies, where important prognostic markers could be targeted for new treatment options. If in the future drugs for multiple targets are available, treatment of dogs with high risk of recurrence could be based on their ACT's molecular malignancy profile, thereby moving towards personalized treatment.

## CONFLICT OF INTEREST

The authors declare no potential conflict of interest.

## Supporting information


**Table S1** Clinical data. Clinical data of the dogs included in this study. Age and body weight indicated in median, with the range in parentheses.

**Table S2** Gene expression correlations with the Utrecht score. Spearman's Rank Order Correlation of mRNA expression levels compared with the Utrecht score as a continuous variable. Significant correlations are flagged with **P < .01, significant *P*‐values are indicated in italic.
