# Supplementary material for: Molecular markers of prognosis in canine cortisol‐secreting adrenocortical tumours
Source: Vet Comp Oncol. 2019 Aug 4;17(4):545–52. doi: 10.1111/vco.12521 (PMC6899893; doi:10.1111/vco.12521)
Supplement: Supplementary file 1 — Table S1 Clinical data. Clinical data of the dogs included in this study. Age and body weight indicated in median, with the range in parentheses. [file VCO-17-545-s001.docx]

**Supplemental Table 1** Clinical data

| **Dogs with adrenocortical tumours (40)** | |
| --- | --- |
| Breed | Jack Russel Terrier (4), Labrador Retriever (4), Dachshund (3), Maltese (2), Schnauzer (2), White Shepherd dog (2), mixed-breed (8), breeds represented once (8) |
| Age | 10 years (2 – 13 years) |
| Body weight | 14 kg (4 – 45 kg) |
| Gender | 19 Female (11 spayed), 21 male (11 neutered) |
| Histopathological Utrecht scores | 0.4, 0.9, 1.1, 1.5, 1.9, 2.3, 2.5, 3.5, 4.0, 4.0, 4.2, 4.4, 4.9, 5.4 (= low risk of recurrence tumours), 6.3, 6.3, 6.4, 6.8, 6.9, 7.1, 7.1, 7.6, 7.7, 8.4, 9.0, 9.1, 9.1, 10.0, 10.3, 10.3, 10.3, 12.4, 13.2, 13.7, 15.4, 18.3, 20.6, 22.1, 25.7, 29.2 (= moderate-high risk of recurrence tumours) |
| **Healthy dogs (11)** | |
| Breed | Beagle (3), mixed-breed (8) |
| Age | 2 years (1 – 5 years) |
| Body weight | 23 kg (10 – 26 kg) |
| Gender | 8 female (none spayed), 3 male (none neutered) |

Clinical data of the dogs included in this study. Age and body weight indicated in median, with the range in parentheses.
