# Supplementary material for: Molecular markers of prognosis in canine cortisol‐secreting adrenocortical tumours
Source: Vet Comp Oncol. 2019 Aug 4;17(4):545–52. doi: 10.1111/vco.12521 (PMC6899893; doi:10.1111/vco.12521)
Supplement: Supplementary file 2 — Table S2 Gene expression correlations with the Utrecht score. Spearman's Rank Order Correlation of mRNA expression levels compared with the Utrecht score as a continuous variable. Significant correlations are flagged with **P < .01, significant P‐values are indicated in italic. [file VCO-17-545-s002.docx]

**Supplemental Table 2** Gene expression correlations with the Utrecht score

| Gene | ***MC2R*** | ***INHA*** | ***SF-1*** | ***VAV2*** | ***PBX1*** | ***VNN1*** | ***SOAT1*** | ***PTTG1*** | ***RRM2*** | ***TOP2A*** | ***MKI67*** | ***CCND1*** | ***RAC1*** | ***BCL2*** |
| --- | --- | --- | --- | --- | --- | --- | --- | --- | --- | --- | --- | --- | --- | --- |
| Correlation coefficient | 0.251 | -0.232 | 0.304 | -0.077 | 0.058 | -0.045 | -0.164 | 0.418** | 0.210 | 0.430** | 0.312 | 0.183 | -0.012 | -0.063 |
| *P*-value (2-tailed) | 0.118 | 0.151 | 0.057 | 0.637 | 0.724 | 0.789 | 0.312 | *0.007* | 0.194 | *0.006* | 0.050 | 0.266 | 0.944 | 0.705 |

Spearman’s Rank Order Correlation of mRNA expression levels compared with the Utrecht score as a continuous variable. Significant correlations are flagged with ** for *P* < 0.01, significant *P*-values are indicated in italic.
